# Long non-coding RNA MALAT1 enhances the protective effect of dexmedetomidine on acute lung injury by sponging miR-135a-5p to downregulate the ratio of X-box binding proteins XBP-1S/XBP-1U

**DOI:** 10.1080/21655979.2021.1967579

**Published:** 2021-09-13

**Authors:** Pengyi Li, Lianbing Gu, Qingming Bian, Dian Jiao, Zeping Xu, Lijun Wang

**Affiliations:** Department of Anesthesiology, Jiangsu Cancer Hospital & Jiangsu Institute of Cancer Research & the Affiliated Cancer Hospital of Nanjing Medical University, Nanjing, Jiangsu, China

**Keywords:** LncRNA MALAT1, DEX, ALI, XBP-1S/XBP-1U, ERS

## Abstract

Acute lung injury (ALI) is the common and clinically severe complication. Dexmedetomidine (DEX) can protect against lipopolysaccharide (LPS)-induced ALI through anti-apoptosis, anti-inflammatory and immune regulatory actions. It is well documented that major causes of LPS-induced ALI are endoplasmic reticulum stress (ERS) and abnormally elevated CHOP. Moreover, XBP-1 can enhance CHOP expression. XBP-1S can aggravate ERS and XBP-1 U can repress ERS. By querying Starbase, miR-135a-5p interacts with XBP-1 and lncRNA MALAT1 sponges miR-135a-5p. It has been reported that MALAT1 interference markedly promoted the apoptosis of pulmonary microvascular endothelial cells in ALI rats by activating TLR4/NF-κB pathway. miR-135a-5p inhibitor remarkably alleviated LPS-induced A549 cell injury through suppressing cell apoptosis. In the present work, LPS was dripped into the nasal cavity of SD rats to establish the rat model of ALI and LPS was also applied to stimulate BEAS-2B cells to imitate ALI *in vitro*. Then, the pathology, lung function indexes, levels of inflammatory factors, apoptosis of lung tissues in SD rats and apoptotic level of BEAS-2B cells were measured, so as to confirm whether upregulation of lncRNA MALAT1 was able to suppress ERS, thus enhancing the protective effect of DEX against ALI. Herein, overexpression of lncRNA MALAT1 strengthened the remission effects of DEX on LPS-triggered ALI, severe pulmonary edema, inflammatory response and cell apoptosis of lung tissues in SD rats and reinforced the anti-apoptosis effect of DEX on LPS-stimulated BEAS-2B cells. Mechanically, lncRNA MALAT1 enhanced the protective effect of DEX against ALI by downregulating the ratio of XBP-1S/XBP-1U to repress ERS.

## Introduction

Acute lung injury (ALI) and its severe form acute respiratory distress syndrome (ARDS) are common and clinically severe complications [1]. The patients are manifested by hypoxemia and decrease in lung compliance [2]. Although in the past few years, the development of intensive care medicine contributes to the significant drop in the incidence and mortality of ALI and ARDS [3]. There is still a lack of clinically effective therapies [4]. Therefore, there is an urgent need to develop appropriate therapeutic drugs or targets to better carry out the precision treatment.

The bacterial endotoxin, lipopolysaccharide (LPS), is a highly proto-inflammatory factor that can trigger systemic inflammation. In lung tissues, LPS is the stimulating factor that induces inflammatory response, resulting in damage and ultimately leading to lung injury. LPS-induced ALI is a widely accepted animal model [5]. Dexmedetomidine (DEX) is an adrenergic receptor agonist with good sedative effect and is widely applied in the process of clinical therapeutics [6]. It has been reported that DEX treatment can protect against LPS-induced ALI through anti-apoptosis, anti-inflammatory and immune regulatory actions [7–9]. However, the specific mechanism of DEX in ALI needs to be further explored.

Non-coding RNA is a type of RNA that cannot encode proteins [10]. At present, accompanied by more extensive researches on microRNAs (miRNAs) and long noncoding RNAs (lncRNAs), increasing studies about the correlations among lncRNAs, miRNAs and ALI pathogenesis are carried out [11,12]. It has been proved that lncRNA MALAT1 was significantly downregulated in ALI rats, and MALAT1 interference markedly promoted the apoptosis of pulmonary microvascular endothelial cells in ALI rats by activating TLR4/NF-κB pathway [13]. Literature also verifies that lncRNA MALAT1 could act as a ceRNA to modulate miR-135a-5p expression [14]. In addition, miR-135a-5p inhibitor remarkably alleviated LPS-induced A549 cell injury through suppressing cell apoptosis [15].

Endoplasmic reticulum can avoid the accumulation of misfolded and unfolded proteins by activating a series of signaling pathways, and then maintain eukaryotic cell homeostasis, which is called endoplasmic reticulum stress (ERS) [16]. It is well documented that major causes of LPS-induced ALI are ERS and abnormally elevated expression of ERS-related C/EBP-homologous protein (CHOP) [17,18]. During the onset and development of ERS, CHOP expression is closely associated with the activation of protein kinase RNA-like ER kinase (PERK)/activating transcription factor 4 (ATF4) and ATF6 pathways [19–21]. In addition, some studies also reveal that another ERS-related X-box binding protein 1 (XBP-1) can enhance CHOP expression [22,23]. The activity and function of XBP-1 are mainly determined by its spliceosome. The changing of nucleotide sequence of XBP-1 mRNA leads to the alternation of amino acid sequence of XBP-1 protein (XBP-1S). XBP-1S can aggravate the unfolded protein response (UPR) and ERS. Additionally, XBP-1 U, which is formed by normal XBP-1 mRNA, can repress UPR and ERS [24,25]. By querying Starbase (http://starbase.sysu.edu.cn/), it is confirmed that miR-135a-5p interacts with normal mRNA of XBP-1, therefore targeting and degrading it. Furthermore, application of DEX could also ameliorate oxidative stress and cell apoptosis in kidney tissues by suppressing ERS [26].

Here, LPS-induced ALI in SD rat model was established and BEAS-2B cells were stimulated by LPS to imitate ALI *in vitro*. The pathology, lung function indexes, levels of inflammatory factors and cell apoptosis of lung tissues in SD rats and apoptosis of BEAS-2B cells were measured, so as to confirm whether upregulation of lncRNA MALAT1 was able to suppress ERS, thus enhancing the protective effect of DEX against ALI.

## Material and methods

### Establishment of rat model of acute lung injury

SD rats were obtained from the Shanghai Laboratory Animal Center of the Chinese Academy of Sciences (Shanghai, China). All experiments were approved and performed according to the guidelines of the Ethics Committee of Jiangsu Cancer Hospital & Jiangsu Institute of Cancer Research & The Affiliated Cancer Hospital of Nanjing Medical University. LPS (7.5 mg/kg) was dripped into the nasal cavity of SD rats to establish the rat model of acute lung injury. 1 h before LPS stimulation, DEX (5 mg/kg; Jiangsu Hengrui Pharmaceutical Co., Ltd., Lianyungang, China) was intraperitoneally injected for lung protection. MALAT1 overexpression lentivirus (Over-MALAT1) and corresponding negative control (Over-NC) were designed by the Genechem (Shanghai, China). SD rats were injected with overexpression lentivirus or control lentivirus 3 d before LPS stimulation. In the current work, the *in vivo* experimental groups were divided into five groups as follows: (I) Control; (II) Non-treated acute lung injury (ALI) rats (Model); (III) ALI rats receiving pre-treatment with DEX; (IV) ALI rats receiving tail vein injection with Over-NC and pre-treatment with DEX; (V) ALI rats receiving tail vein injection with Over-MALAT1 and pre-treatment with DEX. After sacrificing rats, lung tissues were collected for subsequent analysis.

### Determination of lung water content

Lung water content was determined to evaluate the pulmonary edema. SD rats were sacrificed and lungs were collected. Wet weight and dry weight of lungs were measured before and after drying at 80°C for 48 h. Values of lung water content is calculated according to the following formula: lung water content (%) = (wet weight-dry weight)/wet weight × 100%.

### Total protein content and cell counts in bronchoalveolar lavage fluid (BALF)

SD rats were sacrificed and BALF was obtained by injection and retraction of 2 ml PBS thrice. Then, the collected solution was centrifuged at 1,200 × g for 10 min at 4°C. The supernatants were used for determination of total protein content in BALF with bicinchoninic acid (BCA) commercial kits (Beyotime, Shanghai, China). All operations during experiments followed the instructions.

Cell pellet was resuspended in PBS, and cell counts were determined using a hemocytometer following Wright-Giemsa staining for 10 min.

### Enzyme-linked immunosorbent assay (ELISA)

ELISA was performed to detect the levels of inflammatory factors in lung tissues. TNF-α Rat ELISA kit (Abcam, ab100785, MA, USA), IL-1β Rat ELISA kit (Abcam, ab100768, MA, USA), IL-6 Rat ELISA kit (Abcam, ab100772, MA, USA) and MPO Rat ELISA kit (Nanjing Jiancheng Bioengineering Institute, A044-1-1, Nanjing, China) were used for the experiments. All operations of ELISA assays followed the instructions.

### TUNEL staining

Lung tissues of SD rats were sectioned and treated with proteinase K working solution. After that, these slices were washed with PBS thrice. Next, terminal deoxynucleotide transferase (TdT) was incubated with these sections for 15 min. Thereafter, these sections were incubated with TUNEL reaction mixture (Roche, Basel, Switzerland) for 1 h. 4′, 6-diamidino-2-phenylindole (DAPI) was applied for the nuclear staining and incubated in the dark. The fluorescent microscope (Olympus, Tokyo, Japan) was used for acquiring images.

### Lung histopathological analysis

Lung tissues of SD rats were placed in 10% formalin overnight, dehydrated and embedded with paraffin. Then, lung tissues were cut into 5 μm sections. Next, these slices were stained with the hematoxylin and eosin (H&E). In addition, the lung sections were stained with periodic acid-Schiff (PAS). All these sections were observed with the microscope (Olympus, Tokyo, Japan).

### Luciferase reporter assay

Interactions between lncRNA MALAT1 and miR-135a-5p or miR-135a-5p and XBP-1 were assessed via luciferase reporter assay. The amplified lncRNA MALAT1 or XBP-1 3′-UTR sequence and lncRNA MALAT1 MUT or XBP-1 MUT 3′-UTR sequence were inserted into the pGL3 vector (Promega, WI, USA) to construct the luciferase reporter lncRNA MALAT1-WT or XBP-1-WT and lncRNA MALAT1-MUT or XBP-1-MUT. Then, cells were co-transfected with luciferase reporter and miR-135a-5p mimic or mimic NC. The samples were harvested after 48 h and the luciferase activity was evaluated by commercial kits (Promega, WI, USA). Renilla luciferase values were used to normalize Firefly luciferase values. All the operations followed the instructions.

### Cell culture and treatment

BEAS-2B cells were obtained from ATCC (Manassas, VA, USA). Cells were cultured in RPMI-1640 medium (HyClone, UT, USA) supplemented with 10% fetal bovine serum (Gibco, NY, USA) at 37°C in a humidified atmosphere with 5% CO_2_ and 95% air. BEAS-2B cells were incubated with 4 μg/ml LPS and/or 10 μM DEX for 24 h.

### Cell transfection

LncRNA MALAT1 overexpression lentivirus and corresponding negative control were obtained from Genechem (Shanghai, China). LncRNA MALAT1 was overexpressed in BEAS-2B cells by transfecting with the lentivirus. Polybrene (Genechem, Shanghai, China) was applied to enhance the efficiency of transfection according to the manufacturer’s instruction. Briefly, cells (2 × 10^5/^well) were seeded into 24-well plates. 2 µg vectors were mixed with 10 μg/ml polybrene (Santa Cruz Biotechnology, CA, USA) in complete medium. Then, the mixture was added into the plate and transfections were performed at 37°C for 6 h. Subsequently, the medium was replaced with fresh medium and positive cells were selected with 10 μM puromycin (Santa Cruz Biotechnology, CA, USA).

### Cell counting kit-8 (CCK-8) assay

CCK-8 assay was applied for detection of the viability of BEAS-2B cells. Cells were plated into 96 well plates (2 × 10^3^ cells/hole). After 24 h incubation, CCK-8 reagent (Dojindo, Kumamoto, Japan) was added into 96 well plates and cultured for another 2 h at 37°C in the incubator. After that, absorbance of each well at 450 nm was detected using a microplate reader (Bio-Rad, CA, USA).

### Western blotting analysis

Total proteins were extracted using RIPA buffer (Beyotime, Shanghai, China) and the concentration of protein samples was determined via BCA (Beyotime, Shanghai, China) methods. Thereafter, protein samples were separated by 10% SDS-PAGE gel (Beyotime, Shanghai, China) and transferred to PVDF membranes (Millipore, MA, USA). PVDF membranes were blocked in 5% skim milk powder for 1 h and then incubated with primary antibodies at 4 °C overnight. The primary antibodies used in this research were IRE-1 (Abcam, ab37073, 1:2000), CHOP (CST, #5554, 1:1000), XBP-1 U (Abcam, ab37152, 1:2000), XBP-1S (CST, #40,435, 1:1000), Caspase-12 (Abcam, ab62484, 1:1000), Bax (Abcam, ab32503,1:5000), Bcl-2 (Abcam, ab32124, 1:1000), Caspase-3 (Abcam, ab13847, 1:500), Cleaved caspase-3 (Abcam, ab2302, 1:500) and GAPDH (Abcam, ab9484, 1:1000). On the second day, PVDF membranes were washed with PBST for three times and incubated with second antibodies for 2 h at room temperature. At last, protein signals were developed with enhanced chemiluminescence reagents (Pierce, IL, USA) and analyzed with Image J software.

## Reverse transcription-quantitative polymerase chain reaction *(RT-qPCR)*

Total RNA was extracted using Trizol (Thermo Fisher Scientific, MA, USA) methods. Next, mRNA was converted into cDNA with the reverse transcription kit (Roche, Basel, Switzerland). Then, cDNA was amplified with ABI 7500 system (Thermo Fisher Scientific, MA, USA). SYBE Green was used for the detection of fluorescence signal. The sequences of primers were as follows: MALAT1 forward: 5′- GACGGAGGTTGAGATGAAGC −3′ and reverse: 5′- ATTCGGGGCTCTGTAGTCCT −3ʹ; miR-135a-5p forward: 5′- GGCCTCGCTGTTCTCTATGG −3′ and reverse: 5′- GCCACGGCTCCAATCCCTAT −3′; GAPDH forward: 5ʹ- AACGGGAAGCTTGTCATCAATGGAAA −3ʹ and reverse: 5ʹ- GCATCAGCAGAGGGGGCAGAG −3ʹ; U6 forward: 5ʹ- CTCGCTTCGGCAGCACA −3ʹ and reverse: 5ʹ- AACGCTTCACGAATTTGCGT −3ʹ. 2^−∆∆Ct^ was applied for calculation of the relative expressions of target genes [27].

### Statistical analysis

Experimental data in this research was analyzed using Graphpad Prism 7.0 (GraphPad Software Inc., CA, USA) and expressed as mean ± standard deviation (SD). All the experiments were repeated in triplicate. One-way analysis of variance followed by Tukey’s post hoc test was performed to evaluate the differences among diverse groups. The difference was considered as statistically significant when P value was less than 0.05. *p < 0.05, **p < 0.01, ***p < 0.001.

## Results

### Overexpression of lncRNA MALAT1 enhanced the protective effects of DEX on LPS-induced ALI

**Figure 1.**
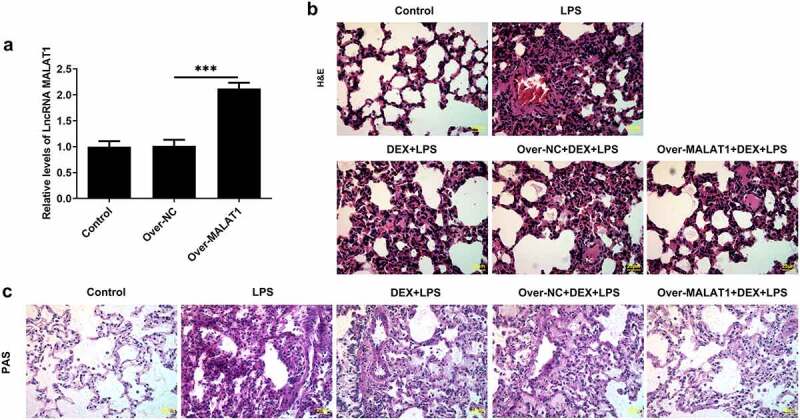
Overexpression of lncRNA MALAT1 enhanced the protective effects of DEX on LPS-induced ALI. (a) SD rats received tail vein injection with MALAT1 overexpression lentivirus or control lentivirus. The efficacy of lentivirus was verified by performing RT-qPCR. (b) H&E staining for lung histopathological analysis and representative images of H&E staining (400×). (c) PAS staining for lung histopathological analysis and representative images of PAS staining (400×)

**Figure 2.**
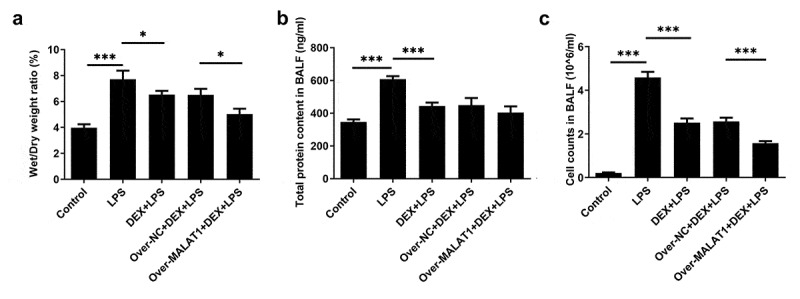
Overexpression of lncRNA MALAT1 enhanced the protective effects of DEX on LPS-induced ALI. (a) Lung water content. (b) Total protein content in BALF. (c) Cell counts in BALF

In order to identify the regulatory function of lncRNA MALAT1 in ALI, SD rats received tail vein injection with MALAT1 overexpression lentivirus or control lentivirus. The efficacy of lentivirus was verified by performing RT-qPCR (Figure 1(a)). HE staining and PAS staining for lung histopathological analysis were performed to determine the effects of lncRNA MALAT1 on LPS-induced ALI. The alveolar structures of control rats are complete, and there is no obvious inflammatory cell infiltration in the lung interstitium and alveolar cavity. In ALI rats, the structure of lung tissue was disordered and mainly manifested as obvious thickening of alveolar septum, infiltration of a large number of inflammatory cells and red blood cells in the lung interstitium and alveolar cavity and partial rupture of alveolar septum. Then, it was observed that DEX therapy relieved the symptoms of lung tissues in ALI rats. Moreover, overexpression of lncRNA MALAT1 further enhanced the therapeutic effects of DEX on ALI (Figure 1(b,c)). Lung water content was determined to evaluate the pulmonary edema. DEX treatment alleviated severe pulmonary edema in ALI rats and overexpression of lncRNA MALAT1 enhanced the remission effect of DEX (Figure 2(a)). In addition, LPS stimulation induced higher levels of total protein content and cell counts in BALF. DEX therapy relieved these symptoms during the development of ALI and upregulation of lncRNA MALAT1 also strengthened the protective role of DEX in ALI (Figure 2(b,c)).

### Overexpression of lncRNA MALAT1 enhanced the relieving effect of DEX on LPS-triggered inflammatory response of lung tissues

**Figure 3.**
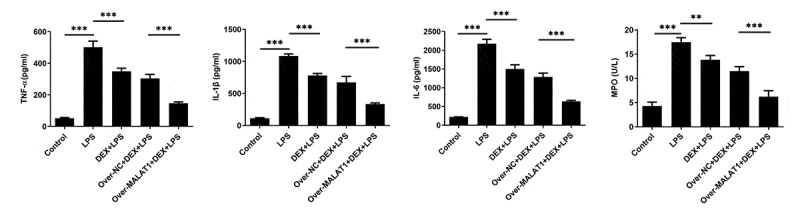
Overexpression of lncRNA MALAT1 enhanced the relieving effect of DEX on LPS-triggered inflammatory response of lung tissues. Levels of TNF-α, IL-1β, IL-6 and MPO in lung tissues were determined with ELISA assays

Inflammatory response is a complex biological reaction during multiple tissue damage. Results from ELISA assays demonstrated that DEX therapy reduced the high levels of TNF-α, IL-1β, IL-6 and MPO in lung tissues of ALI rats. Moreover, the relieving effect of DEX on LPS-triggered inflammatory response of lung tissues was further strengthened after elevation of lncRNA MALAT1 (Figure 3).

### Overexpression of lncRNA MALAT1 enhanced the inhibitory effect of DEX on cell apoptosis in ALI

**Figure 4.**
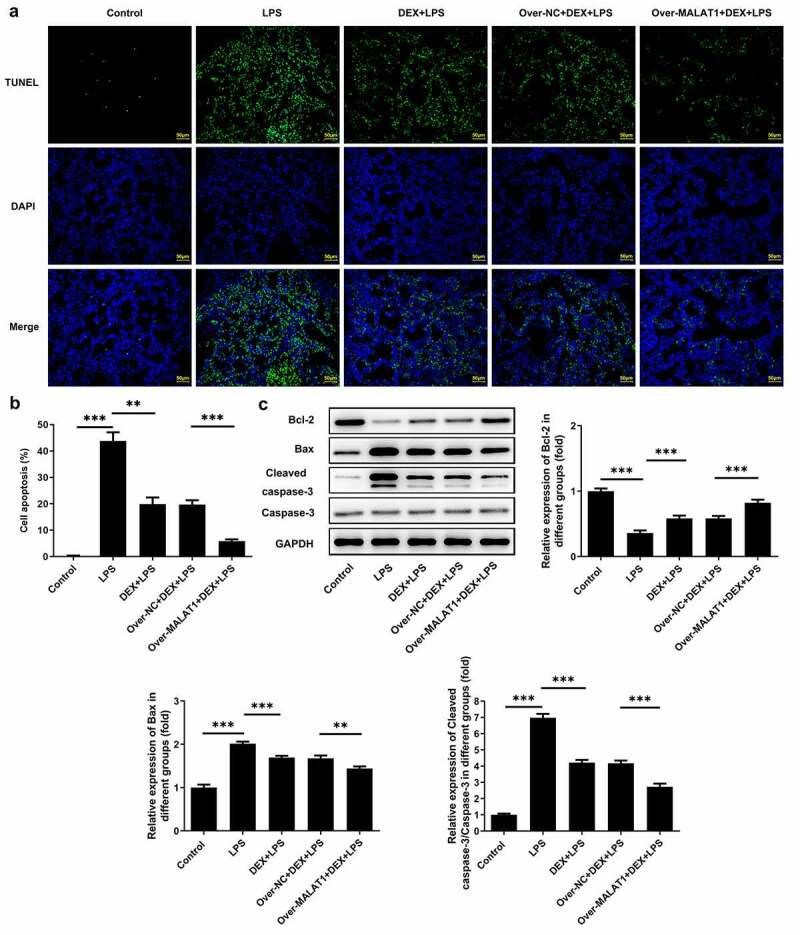
Overexpression of lncRNA MALAT1 enhanced the inhibitory effect of DEX on cell apoptosis in ALI. (a,b) The apoptotic cells in lung tissues were assessed by TUNEL method. (c) Expression levels of apoptosis-related proteins in lung tissues were detected using western blotting assay

Furthermore, TUNEL staining was performed to assess cell apoptosis in lung tissues. In ALI rats, LPS stimulation triggered cell apoptosis in lung tissues, which was hindered after DEX therapy. In addition, overexpression of lncRNA MALAT1 further strengthened the anti-apoptosis effect of DEX (Figure 4(a,b)). After that, levels of apoptosis-related proteins in lung tissues were detected using western blotting assay. It was observed that expression level of Bcl-2 was reduced and expression levels of Bax and Cleaved caspase-3 were elevated after LPS stimulation. DEX therapy recovered Bcl-2 expression and suppressed Bax and Cleaved caspase-3 expression. Additionally, upregulation of lncRNA MALAT1 further strengthened the regulatory effects of DEX on the expression levels of apoptosis-related proteins in lung tissues, suggesting that lncRNA MALAT1 could enhance the anti-apoptosis effect of DEX in ALI (Figure 4(c)).

### LncRNA MALAT1 strengthened the protective effect of DEX on ALI by sponging miR-135a-5p to suppress ERS

**Figure 5.**
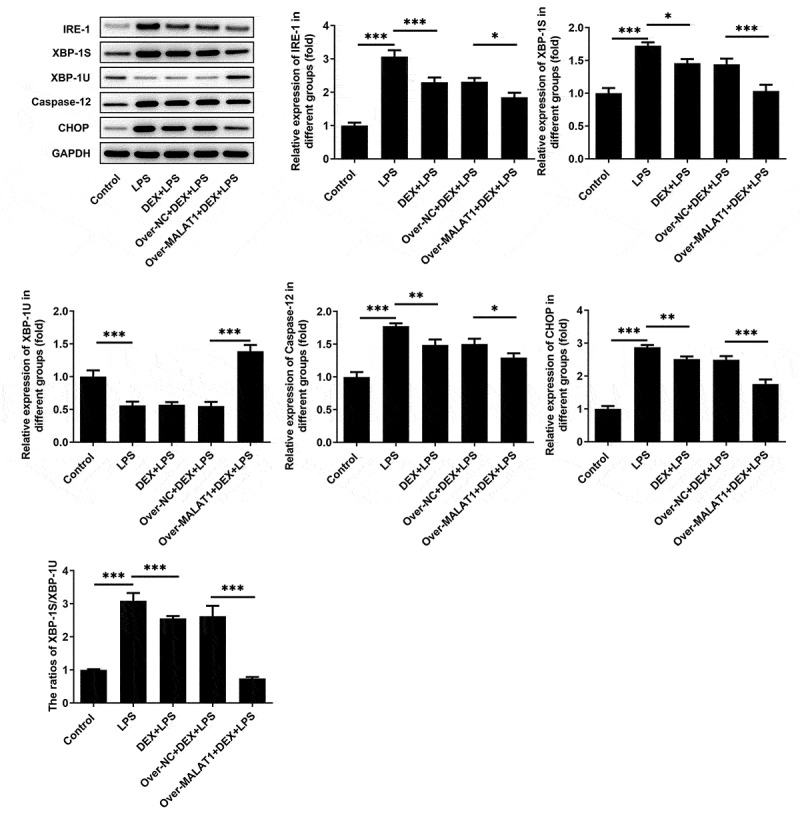
LncRNA MALAT1 could sponge miR-135a-5p to manipulate the ratio of XBP-1S/XBP-1U. (a) The level of lncRNA MALAT1 in lung tissues of SD rats was detected using RT-qPCR. (b) The level of miR-135a-5p in lung tissues of SD rats was detected using RT-qPCR. (c) miR-135a-5p was overexpressed in BEAS-2B cells by transfecting miR-135a-5p mimic and the transfection efficacy was verified via RT-qPCR assay. (d) The predicted complementary binding site of lncRNA MALAT1 and miR-135a-5p. (e) Luciferase reporter assay for assessment of the interaction between lncRNA MALAT1 and miR-135a-5p. (f) The predicted complementary binding site of miR-135a-5p and XBP-1. (g) Luciferase reporter assay for assessment of the interaction between miR-135a-5p and XBP-1

**Figure 6.**
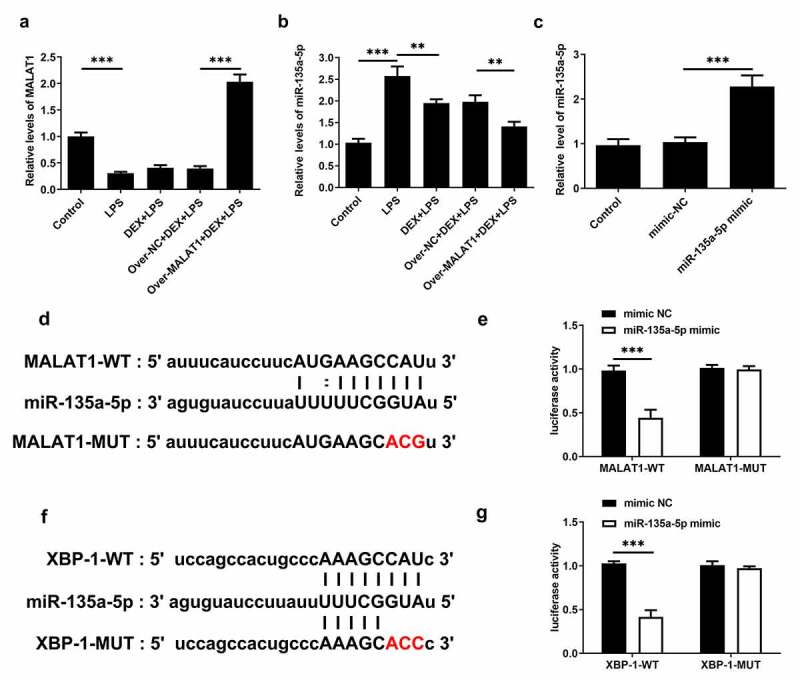
LncRNA MALAT1 sponged miR-135a-5p to suppress ERS. ERS-related proteins including IRE-1, XBP-1S, XBP-1U, Caspase-12 and CHOP in lung tissues of SD rats were determined using western blotting assay. The ratio of XBP-1S/XBP-1U was calculated

In lung tissues of ALI rats, obviously decreased lncRNA MALAT1 expression and increased miR-135a-5p expression were observed. DEX treatment slightly rescued the level of lncRNA MALAT1 and visibly suppressed miR-135a-5p expression. Overexpression of lncRNA MALAT1 enhanced the regulatory effects of DEX on the expressions of lncRNA MALAT1 and miR-135a-5p (Figure 5(a,b)). For subsequent experiments, miR-135a-5p was overexpressed in BEAS-2B cells by transfecting miR-135a-5p mimic (Figure 5(c)). By retrieving Starbase, lncRNA MALAT1 can interact with miR-135a-5p, hinting its potential to sponge miR-135a-5p (Figure 5(d)). Luciferase reporter assay revealed that upregulation of miR-135a-5p distinctly decreased the luciferase activity of MALAT1-WT while had no inhibitory effect on the luciferase activity of MALAT1-MUT (Figure 5(e)). In addition, interaction between miR-135a-5p and XBP-1 was verified by querying Starbase and performing luciferase reporter assay (Figure 5(f,g)). Upregulation of miR-135a-5p significantly decreased the luciferase activity of XBP-1-WT while had no inhibitory effect on that of XBP-1-MUT. These findings prompted that lncRNA MALAT1 could sponge miR-135a-5p to manipulate the expression of XBP-1. Then, expressions of ERS-related proteins in lung tissues were assessed via western blotting analysis. Levels of IRE-1, XBP-1S, Caspase-12 and CHOP were elevated and level of XBP-1 U was reduced after LPS stimulation. The increased ratio of XBP-1S/XBP-1 U was observed in lung tissues of ALI rats. Treatment of DEX restricted the expressions of IRE-1, XBP-1S, Caspase-12, CHOP as well as the ratio of XBP-1S/XBP-1 U. Additionally, overexpression of lncRNA MALAT1 recovered XBP-1 U expression and further suppressed the expressions of IRE-1, XBP-1S, Caspase-12, CHOP as well as the ratio of XBP-1S/XBP-1 U (Figure 6). Hence, we concluded that lncRNA MALAT1 may reinforce the protective effect of DEX against ALI by sponging miR-135a-5p to suppress ERS.

### Overexpression of lncRNA MALAT1 reinforced the anti-apoptosis effect of DEX on LPS-stimulated BEAS-2B cells

**Figure 7.**
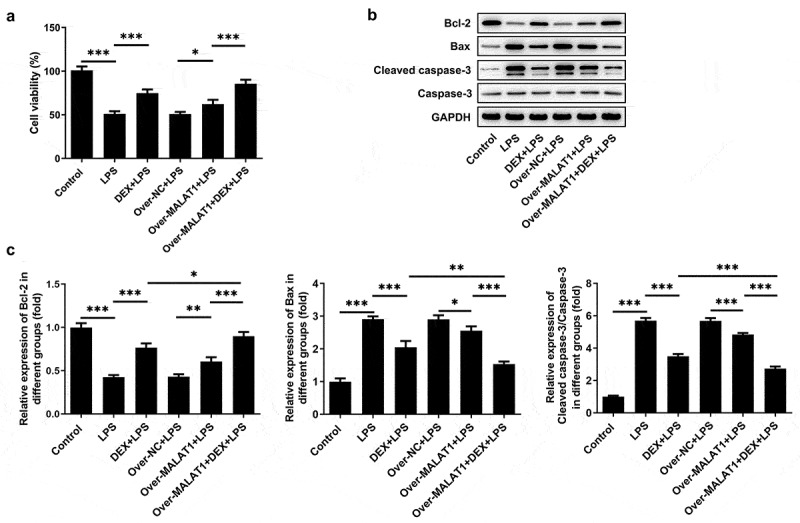
Overexpression of lncRNA MALAT1 reinforced the anti-apoptosis effect of DEX on LPS-stimulated BEAS-2B cells. (a) Cell viability of BEAS-2B cells was determined with CCK-8 assays. (b,c) The expressions of apoptosis-related proteins in BEAS-2B cells were determined with western blotting assay

Furthermore, the biological function of lncRNA MALAT1 was investigated in LPS-stimulated BEAS-2B cells. LPS stimulation suppressed the viability of BEAS-2B cells and application of DEX relieved cell injury caused by LPS. Overexpression of lncRNA MALAT1 further recovered the viability of LPS-stimulated BEAS-2B cells, enhancing the protective effect of DEX (Figure 7(a)). Next, the expressions of apoptosis-related proteins were determined with western blotting assay. LPS stimulation reduced the level of Bcl-2 and elevated the levels of Bax and Cleaved caspase-3 in BEAS-2B cells, which were partly reversed by DEX therapy. In addition, overexpression of lncRNA MALAT1 further increased Bcl-2 expression and decreased the expressions of Bax and Cleaved caspase-3, strengthening the anti-apoptosis effect of DEX on LPS-stimulated BEAS-2B cells (Figure 7(b,c)).

### LncRNA MALAT1 enhanced the protective effect of DEX on LPS-stimulated BEAS-2B cells by manipulating IRE-1/XBP-1 pathway to suppress ERS

**Figure 8.**
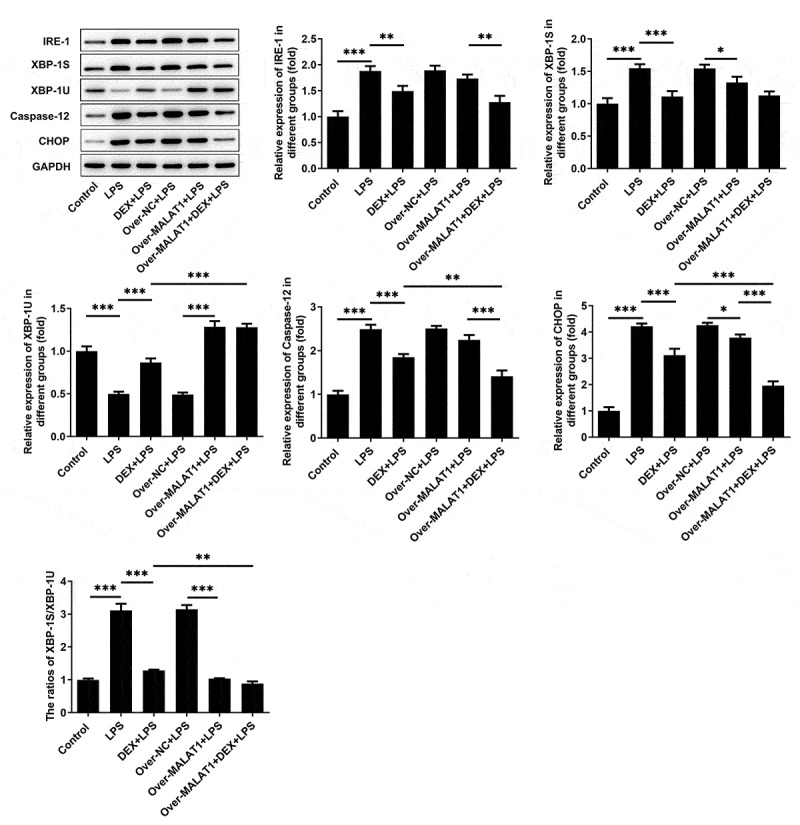
LncRNA MALAT1 enhanced the protective effect of DEX on LPS-stimulated BEAS-2B cells by manipulating IRE-1/XBP-1 pathway to suppress ERS. ERS-related proteins including IRE-1, XBP-1S, XBP-1U, Caspase-12 and CHOP in BEAS-2B cells were determined using western blotting assay. The ratio of XBP-1S/XBP-1U was calculated

Meanwhile, expressions of IRE-1, XBP-1S, XBP-1 U, Caspase-12 and CHOP in BEAS-2B cells were determined using western blotting assay. In LPS-stimulated BEAS-2B cells, obviously increased expressions of IRE-1, XBP-1S, Caspase-12, CHOP as well as elevated ratio of XBP-1S/XBP-1 U and decreased expression of XBP-1 U were observed. DEX therapy suppressed IRE-1, XBP-1S, Caspase-12, CHOP as well as the ratio of XBP-1S/XBP-1 U and rescued XBP-1 U, thereby repressing ERS. Importantly, overexpression of lncRNA MALAT1 reinforced the regulating effects of DEX on IRE-1/XBP-1 pathway and then further suppressed ERS to protect against cell injury caused by LPS (Figure 8).

## Discussion

ALI is a severe lung disease. Although several progress has been achieved in the prevention and treatment, the mortality rate of ALI still remains high [2,4]. At present, there are plenty of literature reports focusing on the protective effect of DEX on LPS-induced ALI [7–9]. Here, in this current work, LPS was dripped into the nasal cavity of SD rats to establish the rat model of ALI and LPS was also applied to stimulate BEAS-2B cells to imitate ALI *in vitro*. The structures of lung tissues in ALI rats were disordered and mainly manifested as obvious thickening of alveolar septum, infiltration of a large number of inflammatory cells and red blood cells in the lung interstitium and alveolar cavity and partial rupture of alveolar septum. ALI rats emerged with severe pulmonary edema, higher levels of total protein content and cell counts in BALF and obvious cell apoptosis of lung tissues. DEX therapy relieved these symptoms during the development of ALI. In addition, LPS stimulation suppressed the viability of BEAS-2B cells and application of DEX alleviated cell injury caused by LPS.

There is increasing evidence revealing that LPS-induced lung injury is mainly due to elevated expression of CHOP and occurrence of ERS [17,18]. The occurrence and development of ERS are associated with expressions of ATF-4, ATF-6 and IRE-1 [21,28]. IRE-1 can increase the ratio of XBP-1S/XBP-1 U, thus enhancing CHOP expression and accelerating the development of ERS [29,30]. Additionally, higher levels of XBP-1S can induce UPR in multiple types of cells, therefore leading to the enhancement of CHOP and occurrence of ERS [31]. Furthermore, CHOP expression is elevated during the development of LPS-induced lung injury [32]. In the present study, increased expressions of IRE-1, XBP-1S, Caspase-12, CHOP as well as the ratio of XBP-1S/XBP-1 U were observed in lung tissues of ALI rats and LPS-stimulated BEAS-2B cells. Moreover, literature revealed that DEX could alleviate cerebral ischemia reperfusion injury by suppressing the expressions of ERS-related proteins (GRP78, p-PERK, CHOP and Cleaved caspase-12) [33]. Herein, elevated levels of IRE-1, XBP-1S, Caspase-12, CHOP as well as the ratio of XBP-1S/XBP-1 U in lung tissues of ALI rats and LPS-stimulated BEAS-2B cells were reversed following DEX therapy, suggesting that DEX could alleviate LPS-induced ALI *in vivo* and *in vitro* by suppressing ERS.

Furthermore, by retrieving Starbase, it was verified that miR-135a-5p could interact with XBP-1 and therefore repress the expression of XBP-1 U. A study has also reported that miR-135a-5p could aggravate LPS-induced ALI by inducing cell apoptosis [14]. Additionally, some studies have reported that miR-135a-5p could markedly induce tissue damage and fibrosis [34,35]. Results of our current study supported that downregulation of miR-135a-5p reduced the level of XBP-1S and elevated the level of XBP-1 U, thereby suppressing ERS.

LncRNAs are RNA molecules consisting of more than 200 nucleotides. Increasing studies have confirmed that lncRNAs are frequently involved in multiple types of physiological processes such as cell differentiation, apoptosis and inflammatory responses [36,37]. Importantly, literature reveals that lncRNA MALAT1 can relieve the symptoms of sepsis-induced acute lung injury via TLR4/NF-κB and p38 MAPK signaling pathways [13]. In this study, we disclosed that overexpression of lncRNA MALAT1 reinforced the remission effects of DEX on severe pulmonary edema, inflammatory responses and cell apoptosis of lung tissues in ALI rats, enhancing the protective effects of DEX on LPS-induced ALI. Through browsing Starbase, it was verified that lncRNA MALAT1 possessed the potential to sponge miR-135a-5p. Besides, there was a research reporting that lncRNA MALAT1 served as a molecular sponge for miR-135a-5p [14]. Luciferase reporter assay showed that lncRNA MALAT1 could interact with miR-135a-5p and negatively regulate miR-135a-5p expression to restrict the biological effect of miR-135a-5p. Next, it was also discovered that overexpression of lncRNA MALAT1 enhanced the inhibitory effects of DEX on the expressions of IRE-1, XBP-1S, Caspase-12, CHOP as well as the ratio of XBP-1S/XBP-1 U. All results above prompted that lncRNA MALAT1 may strengthen the protective effect of DEX on ALI by sponging miR-135a-5p to suppress ERS. Consistent with the findings in the rat model of ALI, lncRNA MALAT1 was proved to enhance the protective effect of DEX on LPS-stimulated BEAS-2B cells by manipulating IRE-1/XBP-1 pathway to suppress ERS.

## Conclusion

To sum up, overexpression of lncRNA MALAT1 strengthened the remission effects of DEX on LPS-triggered ALI, severe pulmonary edema, inflammatory response and cell apoptosis of lung tissues in SD rats. Moreover, overexpression of lncRNA MALAT1 reinforced the anti-apoptosis effect of DEX on LPS-stimulated BEAS-2B cells. Mechanically, lncRNA MALAT1 downregulated the ratio of XBP-1S/XBP-1 U to repress ERS, thereby enhancing the protective effect of DEX against ALI. These findings highlighted the protective property of lncRNA MALAT1 in ALI and may provide new strategies for ALI therapy.

## Data Availability

Datasets analyzed during the current study are available from the corresponding author on reasonable request.
